# A Central School of Medicine for London

**Published:** 1901-12-28

**Authors:** 


### A Central School of Medicine for London.

Driven apparently by the hard fact, which has now become patent to all, that London is rapidly ceasing to attract medical students, the Faculty of Medicine of the University of London has presented a report to the Senate on the concentration of preliminary and intermediate teaching which, if acted on, will probably do much to improve the teaching of these subjects. The facts which were brought before the Faculty of Medicine were of sufficient gravity, for by comparing two periods of five years each, namely from 1879 to 1883, and from 1896 to 1900, respectively, it appeared that in the latter period there had been a total falling off in the London entries of 878, or an average of 175 entries per annum, while in the provincial entries there had been a total increase of 596, or an average increase of 120 entries a year. That shows where the students are going, and, as we pointed out a few weeks ago, we all know why; the reason being that they go where they can get a degree. What the Faculty of Medicine has done is to recommend that the Senate should take steps to secure funds to enable it to establish in the near neighbourhood of the University a school of preliminary and intermediate medical studies. The faculty, moreover, states that it has no doubt of the immediate success of such a school if ample educational facilities and adequate remuneration for the teachers can be provided without increasing the present cost of medical education to the students; if arrangement be made for teaching all classes of students, whether preparing for the examinations of the University or of other qualifying bodies, so that any of the existing schools which chose to do so could at once abandon the teaching of the preliminary and intermediate subjects; and if adequate provision be made the instruction of women students under the direct control of the University. All this is very satisfactory so far as it goes, and there can be little doubt that the advantages placed at the disposal of the London student will be increased by the proposed concentration of teaching. But it is not the lack of instruction so much as the lack of a degree at the end of it that drives away the students, and we doubt whether anything that is done in the way of improving the teaching will prove effectual in turning the stream of students back again Londonwards unless it be coupled with such alterations in the regulations and examinations of the University as shall throw open to the ordinary student the opportunity of obtaining a pass degree on the same terms as exist at other universities. This is entirely a matter of competition between the several universities in meeting a definite want, and although we would all be glad, when we have once got the students, to see competition in teaching so keen as to render the instruction to be obtained in London superior to that which can be got elsewhere, the first thing is to get the students, and for that purpose we must give them the same reward as they obtain at other universities. In regard to all this it must be remembered that the medical students of the University of London are on quite a different footing from the candidates for mere Arts degrees. These good people are at no great loss if they miss an examination. No residence being required, they may study at home at their own time; if they fail once they may try again, and except for the waste of time they are none the worse. But with medical students residence in connection with a recognised school is essential, a certain clearly defined course of study has to be gone through, and in default of sufficient means by which to support himself at his school for another year, failure at an examination may involve a man in total failure and deprivation of his degree. One would like to see all those who study medicine in London attached in some way to the University, so that if they turned out well and made their mark they might finish with a degree. As it is the matriculation keeps out of the University all except those who have been so well taught at school as to be able to matriculate; the reputed severity of the further examinations drives those who wish for a degree to enter at other universities; while the regulations make it impossible for those men—a very considerable class—who only begin to feel their powers when they are half through their curriculum, to join the University without going back again to their school studies. The University should be made attractive and useful not to selected students only, but to all; the degree should be accessible not to precocious geniuses alone, but to average men; and then its honours, its "first classes," its gold medals, and other insignia of superiority may be struggled for by the elect to their hearts' content. When things are arranged on such a basis, when a great central school is opened for the preliminary and intermediate studies, and when the hospitals and their attached schools are left to devote themselves to purely professional training, then London will become a great centre for medical teaching. At present its position is gently waning.

